# Synovial Chondromatosis of Hip Joint in a Patient with Ankylosing Spondylitis: A Case Report

**DOI:** 10.31729/jnma.7370

**Published:** 2022-05-31

**Authors:** Ajay Kumar Yadav, Sujan Raj Paudel, Dilip Kumar Yadav

**Affiliations:** 1 Department of Orthopaedics and Trauma Surgery, Scheer Memorial Adventist Hospital, Banepa, Kavre, Nepal

**Keywords:** *ankylosing spondylitis*, *case reports*, *hip joint*, *synovial chondromatosis*

## Abstract

Synovial chondromatosis in association with ankylosing spondylitis is extremely rare and has been reported only once before and this case report is presenting a similar case. The knee is the preferential site of involvement with involvement of the hip being reported sparsely. We herein report a case of a 52-year-old male who came with complaints of the lower back pain for 5 years and left hip pain for 1.5 years who was diagnosed with synovial chondromatosis of the hip joint with axial ankylosing spondylitis and was managed operatively. We here review briefly the clinical manifestations, pathogenesis, diagnosis, previously reported cases as well as treatment of synovial chondromatosis in patients with immune-mediated inflammatory arthritides. There should be a high index of suspicion to diagnose synovial chondromatosis in association with inflammatory arthritides. We also believe that surgical management is an effective method of treatment of an established synovial chondromatosis of the hip joint.

## INTRODUCTION

Synovial chondromatosis is an uncommon benign clinical entity characterised by metaplastic proliferation of the synovial membrane with the formation of osteocartilaginous loose bodies.^1^ It is mostly monoarticular and can occur in a joint without pre-existing disorder referred to as primary synovial chondromatosis but can also be seen in joints with an underlying disease called secondary synovial chondromatosis.^2^ Secondary synovial chondromatosis has also been reported in association with immune-mediated inflammatory diseases such as Rheumatoid Arthritis (RA) and Relapsing Polychondritis (RP).^2,3^ However, synovial chondromatosis has been reported only once before in association with ankylosing spondylitis to the best of our knowledge.^4^ We hereby report a 52-year-old male who was diagnosed with synovial chondromatosis of the hip joint with axial ankylosing spondylitis and was managed operatively.

## CASE REPORT

A 52-years-male presented with complaints of low back pain with morning stiffness for the past 5 years with pain in the left hip and limping for 1.5 years. However, his main concern was his left hip joint pain. He did not have a history of trauma before the onset of his complaints. Regarding his past history, he was diagnosed with musculoskeletal Tuberculosis (TB) 8 years back for which he took Antituberculosis Therapy (ATT) for 8 months and was declared cured. He was also diagnosed with ankylosing spondylitis but was on medication on and off for it. He was better when he was on medication. He did not give any significant family history. He is a farmer by occupation. On physical examination, he had an antalgic gait, restriction of lumbar spine movement, gross wasting of thigh muscles, swelling in the right hip joint, a moderate rise of skin temperature over the left hip joint and local tenderness at greater trochanter and anterior joint line of the left hip joint. Moreover, the left greater trochanter, patella and malleoli were comparatively at a higher level compared to the normal limb. Flexion at hip was possible upto 30^o^, abduction and adduction were 10^o^ each. The hip could be internally rotated up to 15^o^. However, extension and external rotations were completely restricted. Patrick's sign was found to be positive. Harris's hip score at admission was 63.

**Figure 1:**
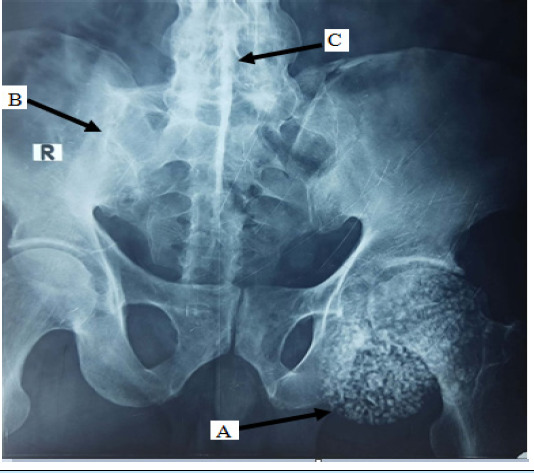
X-ray showing A) Preoperative radiograph of the right hip joint with features of synovial chondromatosis, B) Fused right-sided sacroiliac joint, and C) 'Dagger' signs which are suggestive of ankylosing spondylitis.

**Figure 2:**
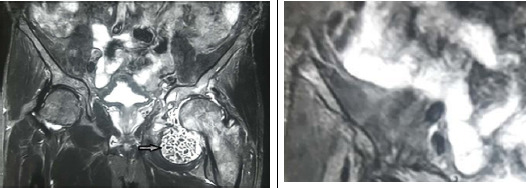
X-ray showing preoperative magnetic resonance imaging of hip joints showing in numerous loose bodies as shown by arrow suggestive of synovial chondromatosis.

His haemoglobin was 15.6 g/dl, random blood sugar 98 mg/dl, serum alkaline phosphatase 80 U/l, human immunodeficiency virus was negative, hepatitis B surface antigen was negative, hepatitis C virus was negative. The X-ray revealed multiple radiopaque joint bodies which were small and uniform. Complete fusion of the right sacroiliac joint with partial fusion of the left sacroiliac joint was also noted. There was a fusion of L5-S1 disc space. Diagnosis of synovial chondromatosis with ankylosing spondylitis was made on the basis of X-ray imaging (Figure 1) and was confirmed by magnetic resonance imaging (Figure 2).

**Figure 3:**
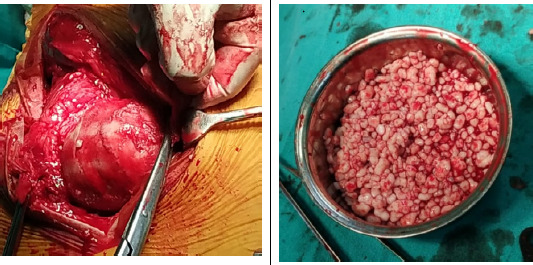
Intraoperative images demonstrating surgical dislocation of hip and loose bodies.

He was managed with surgical hip dislocation under spinal anaesthesia. Synovectomy and removal of loose bodies were performed (Figure 3).

**Figure 4:**
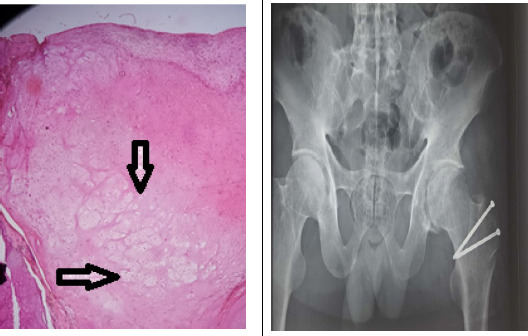
Immediate postoperative X-ray of hip joints and microscopic pictures showing numerous lobules of hyaline cartilage consisting of chondrocytes along with areas of ossification as shown by arrows.

Post-operative X-ray showed removal of loose bodies. Microscopic examination of the sample showed numerous lobules of hyaline cartilage consisting of chondrocytes along with areas of ossification and thus the patient was diagnosed with secondary synovial chondromatosis of the hip joint (Figure 4).

Post-operatively the patient was mobilized with nonweight bearing for 6 weeks with avoidance of active abduction and extreme flexion or rotation of the hip. He was kept on indomethacin 25 mg thrice a day for 6 weeks and sulfasalazine 500 mg twice a day. At 6 weeks, weight-bearing was allowed with progressive muscle strengthening around hip and knee joints.

At 6 months follow up there were significant improvements in the movements of the left hip. About 45° of flexion at the hip was noted. Abduction and adduction increased to 30^o^, internal rotation to 45^o^ and external rotation was increased up to 10^o^. His Harris hip score at 6 months was 76. An X-ray was performed which showed a good union of greater trochanter osteotomy site and no recurrence of his disorder. He had occasional complaints of low back pain or morning stiffness at this stage.

**Figure 5:**
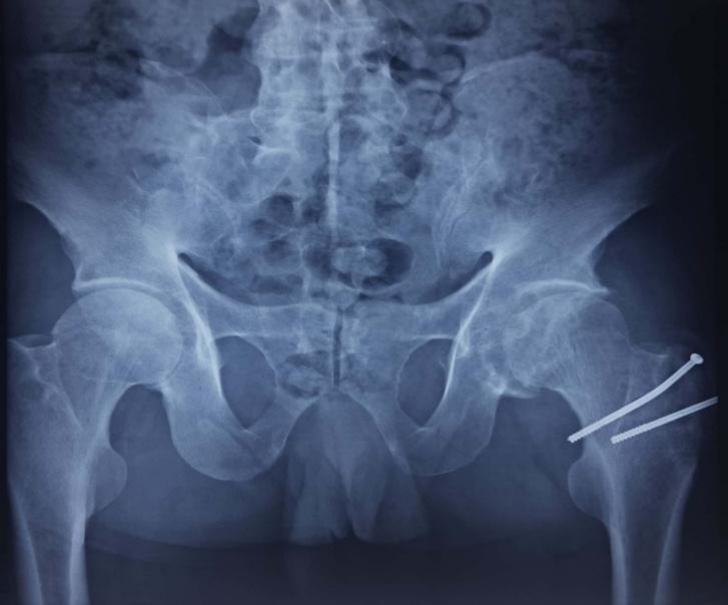
X-ray of the hip at 1 year follow up.

Similarly, at a 1-year follow-up visit, 60^o^ of hip flexion was noted. However, there was no change in the abduction, adduction and internal rotation as compared to 6 months follow-up visit but external rotation increased up to 30^o^. The patient mentioned a significant improvement in low back pain and morning stiffness. Harris's hip score was 82 and the X-ray showed a good union of greater trochanter osteotomy site and no recurrence of his disorder. However, loosening of the screw was noted in the X-ray and the patient was advised for implant removal which he refused (Figure 5).

## DISCUSSION

There is no consensus on the actual prevalence of primary or secondary synovial chondromatosis. Primary synovial chondromatosis occurs when diseased synovium produces nodules that are osteocartilaginous which eventually detach and become loose bodies whereas secondary synovial chondromatosis occurs due to chondrocyte shedding in the setting of trauma, osteoarthritis, and avascular necrosis.^1^

The exact aetiology of the disease is not known. There have been many theories, but none of them so far have been able to pinpoint an exact aetiology. The pathogenesis of synovial chondromatosis has been presumed to be reactive where there is the metaplastic transformation of synovial cells into chondrocytes via an unknown stimulus.^5^ One of the studies stated that there is a fairly low risk of malignant transformation (5%), but is much higher than in well-organized bone diseases like Paget's disease.^5^

Patient can present asymptomatically or may present with joint pain, reduced range of motion, swelling, repeated effusions, crepitus, locking and loose bodies symptoms such as palpable mass and tenderness at the joint line.^1^ The diagnosis is mostly delayed until a more advanced stage of the disease has been reached when symptoms such as pain, swelling and loss of joint movement occur. It can sometimes recur and malignant transformation has also been reported rarely. Synovial chondromatosis was classified by Milgram into three phases as per the histological studies: phase I: an intrasynovial disease with no loose bodies; phase II: transitional lesions with both active intrasynovial proliferation and free loose bodies; and phase III: multiple free osteochondral bodies with no demonstrable intrasynovial disease.^6^ In our case it was Milgram phase II.

The radiographic findings depend on the degree of calcification within the cartilaginous bodies. However, definitive diagnosis based on the observation of loose bodies in plain radiography can be difficult.^7^ Ultrasound generally shows a well-circumscribed peri-articular or intra-articular hypoechoic mass which contains multiple echogenic foci. Ossified loose bodies not otherwise visualized on plain radiographs can be seen in Computed Tomography (CT) scans and can sometimes show erosions as well if present. A study has recommended Magnetic Resonance Imaging (MRI) as a more sensitive diagnostic study than other studies.^8^ MRI has the advantage that it can delineate cartilage clearly and can show the cartilaginous bodies which appear as signal voids on all sequences and are sometimes surrounded by bright signal intensity on T2-weighted images. Technetium bone scans through nonspecific can be of some benefit in a few cases.

If the symptoms are not relieved by conservative treatments, surgical intervention is recommended for symptomatic patients with synovial chondromatosis of the hip.^8^ Complete synovectomy has been found to reduce the rate of recurrence.^1^ Another major concern besides recurrence is the malignant transformation. There is a great diagnostic dilemma both for the radiologist as well as the pathologist in diagnosing chondrosarcoma arising from chondromatosis because of similar features between synovial chondromatosis and low-grade sarcoma.^9^

In a few reported cases of synovial chondromatosis with inflammatory arthritis, invasive treatment was mostly given when a single joint was involved.^2-4^ However, in another study, synovial chondromatosis of the knee associated with axial ankylosing spondylitis, management was done with the biologic agent etanercept which provided long-term symptomatic relief but there was no reduction in the number of loose bodies at two years follow up.^4^

Open, as well as arthroscopic synovectomy and removal of loose bodies, have been described for the surgical treatment of synovial chondromatosis. In a study, comparing arthroscopic and open synovectomy in synovial chondromatosis, it was concluded that the arthroscopic procedure can be opted in the initial stages of the disease, whereas open if joint involvement is greater.^10^ In cases where there is concomitant synovial chondromatosis with well-established loose bodies (Milgram phase II /III) along with inflammatory arthritis, management should include complete surgical removal of loose bodies and complete synovectomy along with management of inflammatory arthritis. Since our case was Milgram phase II, we opted for surgical hip dislocation with open synovectomy and complete removal of loose bodies. The patient was also offered an alternative treatment option of total hip replacement but could not offer it financially.

Even though there is complete symptomatic relief in our patient with surgery and medical management, he will require close and long-term follow-up to determine recurrence, functional impairment of the hip as well as malignant transformation. The limitation of this study is that this study has only one case and the follow-up period is less.

Secondary synovial chondromatosis is a very rare entity and not many cases have been reported so far. There is a paucity of articles related to such conditions. There needs to be a high index of suspicion in order to find the concurrent occurrence of such conditions and especially the concomitant occurrence of synovial chondromatosis and ankylosing spondylitis.
